# The Use of mHealth in Orthopedic Surgery: A Scoping Review

**DOI:** 10.3390/ijerph182312549

**Published:** 2021-11-28

**Authors:** Sara Dionisi, Noemi Giannetta, Emanuele Di Simone, Francesco Ricciardi, Gloria Liquori, Aurora De Leo, Lorenzo Moretti, Christian Napoli, Marco Di Muzio, Giovanni Battista Orsi

**Affiliations:** 1 Department of Biomedicine and Prevention, University of Rome Tor Vergata, 00133 Rome, Italy; sara.dionisi@uniroma1.it (S.D.); gloria.liquori@gmail.com (G.L.); aurora.deleo@uniroma1.it (A.D.L.); 2 Faculty of Philosophy, Vita-Salute San Raffaele University, 20132 Milan, Italy; noemi.giannetta@uniroma1.it; 3 Nursing, Technical, Rehabilitation, Assistance and Research Department, IRCCS Istituti Fisioterapici Ospitalieri—IFO, 00144 Rome, Italy; emanuele.disimone@uniroma1.it; 4 Department of Clinical and Molecular Medicine, Sapienza University of Rome, 00185 Rome, Italy; ricciardi.francesco@outlook.com (F.R.); marco.dimuzio@uniroma1.it (M.D.M.); 5 Orthopaedics and Traumatology, Azienda Ospedaliera Universitaria Consorziale Policlinico di Bari, 70124 Bari, Italy; lorenzo.moretti@libero.it; 6 Department of Surgical and Medical Sciences and Translational Medicine, Sapienza University of Rome, 00185 Rome, Italy; christian.napoli@uniroma1.it; 7 Department of Public Health and Infectious Diseases, Sapienza University of Rome, 00185 Rome, Italy

**Keywords:** mHealth, mobile application, orthopedic surgery

## Abstract

(1) Background: It is well known that the success of surgical procedures is related to optimal postoperative management and follow-up. In this regard, mHealth technologies could potentially improve perioperative care. Based on these considerations, the objective of this scoping review is to evaluate the current status and use of mHealth interventions designed to provide perioperative care in orthopedic surgery. (2) Methods: This scoping review was conducted in accordance with the PRISMA statement (Extension for Scoping Review) and follows the framework of Arskey and O’Malley. (3) Results: The use of mHealth in the surgical setting is mainly oriented towards the development of applications for monitoring post-operative pain and optimizing communication between the various health professionals involved in patient care. (4) Conclusions: The mHealth systems can have a positive impact both on patient participation in the therapeutic process and on the communication between health professionals, increasing the quality of care.

## 1. Introduction

eHealth, or electronic health, represents the application of Information and Communication Technologies (ICT) to health and health care systems [1] Its development is the result of the analysis of different aspects such as: an increasing demand for health services, and the need to provide the best health care under conditions of limited economic resources. Other relevant aspects supporting these innovative applications are the need of limiting adverse events and improved well-being in the citizens and managing massive amounts of health information available promptly and safely, to conduct effective treatment. The use of this service includes several systems: electronic records, electronic prescriptions, telemedicine services for remote patient care, health information systems, and the mobile health.

In this context, the use of mobile wireless technologies for public health, also called mHealth, is defined as “all medical and public health practice supported by mobile devices, such as mobile phones, or those for patient monitoring, personal digital assistants (PDAs), and other wireless devices. mHealth involves the use and capitalization on a mobile phone’s core utility of voice and short messaging service (SMS) as well as more complex functionalities telecommunications (3G and 4G systems), global positioning system (GPS), and Bluetooth technology” [2]. Several studies and literature reviews show that the use of digital health intervention is useful to manage long-term health conditions or medication adherence and to improve postoperative outcomes [3,4,5,6,7]. In addition, other studies [8,9] show how the use of mHealth can improve patient empowerment and participation in the care process, being an engaging and stimulating educational tool.

Moreover, the use of new technologies has been reported as challenging during health emergencies relate to infectious disease, such as COVID-19. As a matter of fact, the use of this practice may reduce direct medical contact and, therefore, occasion of contagion [10].

Specifically, it is well known that the success of adult and pediatric surgery procedures is linked to an optimal postoperative management and follow-up. At this stage, mHealth technologies could potentially improve preoperative and postoperative care [11]. This is confirmed by several studies in all fields of surgery such as cardiac surgery [12], bariatric surgery [13], and gastrointestinal surgery [14]. To this date, there have not been scoping reviews that show the advantages or disadvantages of the utilization of mHealth technologies in orthopedic surgery. Orthopedic surgery is the field of medicine dealing with problems affecting the musculoskeletal system. Major surgeries are performed on the ankle, knee, hip, wrist, elbow, shoulder, and spine, and usually require long recovery times spent in treatment and rehabilitation facilities. Today, orthopedic surgeons face many new challenges than in the past, especially due to both the increased amount of care required and a decreasing surgeon workforce [15]. Moreover, patients want to spend much more time out of hospital and/or rehabilitation facilities.

In this scientific context, objectives of this scoping review are: (i) to evaluate the current state and use of mHealth interventions designed to provide perioperative care (pre, intra, and post) in orthopedic surgery; and (ii) to make available an overview of the main interventions which, through the use of mHealth, can improve the management of the perioperative pathway for both patients and professionals.

## 2. Materials and Methods

### 2.1. Literature Search

This scoping review was conducted in accordance with the PRISMA statement (extension for Scoping Review) [16] and follows the framework of Arskey and O’Malley [17] (Supplementary Materials File 1).

The decision to conduct a scoping review was related to the aim to explore in general the phenomenon under study [18], which appears to be from preliminary research of recent interest in the context of orthopedic surgery. According to Arskey and O’Malley’s framework [17], there are five main phases of a scoping review: 1. Definition of the research question, 2. Identification of the pertinent study; 3. Selection of the studies; 4. Data classification; 5. Comparison, summary, and presentation of the results.

### 2.2. Step 1: Definition of the Research Question

“What are the main mobile applications used in the orthopedic surgery setting in order to improve the management of the perioperative care, both for professionals and patients?”

### 2.3. Step 2: Identification of the Pertinent Study

The protocols used to construct the search string were population (all health professionals and patients), intervention (use or implementation of the mHealth), outcome (benefits to patients and professionals), and setting (orthopedic surgery). All mHealth and orthopedic surgery synonyms were chosen and combined with “OR” and “AND” Boolean operators. The search string can be found in Table 1. The search string was developed by two analysts (EDS, NG) after consulting the main terms used in the different databases chosen. The search was conducted on the following databases: PubMed, Cumulative Index to Nursing and Allied Health Literature (CINAHL), Psychological Abstracts Information Services (PsycINFO), ERIC, and National Library of Medicine (MEDLINE) via EBSCO and Cochrane. In order to investigate the survey phenomenon as fully as possible, no time limits were placed as the databases themselves return articles from 2014 onwards. Finally, only studies published in English and Italian were considered. Language was not placed as a filter in the databases, but articles were evaluated individually, to find, where available, an English version of studies found in other languages. We chose to consider only articles published in English or Italian for two main reasons: The first is due to the linguistic knowledge of the authors who mastered Italian and English; the second reason is related to the fact that English is the main language used in international scientific publications.

### 2.4. Step 3: Selection of the Study

The results obtained from the different databases were imported to the Medline^®^ bibliographic management software and duplicates were removed.

The following criteria were posed for study selection:

Inclusion criteria

- All studies where the target population is health professionals (physicians, nurses, radiology technicians) and/or patients;
- Studies that address the use, development, and implementation of mHealth systems, and reporting the benefits to both professionals and patients in using mHealth tools;
- Studies focusing on the setting of orthopedic surgery and perioperative period. Specifically, studies focusing on musculoskeletal surgery will be included (e.g., surgeries performed on the ankle, knee, hip, wrist, elbow, shoulder, and spine);
- Observational, cross sectional, experimental, and quasi-experimental studies.

Exclusion criteria

- Studies dealing with the use of systems other than mHealth (such as computerized records or artificial intelligence systems);
- Studies focusing on settings other than orthopedic surgery (e.g., medical, pediatric) or involving home and/or community-based care;
- Literature reviews (systematic reviews, narrative reviews, umbrella reviews, etc.), qualitative studies, and the gray literature.

Based on the selected criteria, the first stage of screening was performed by two independent analysts (SD, FR), through reading the title and abstract, to define relevant articles. Articles deemed doubtful were analyzed by reading the full text in the next eligibility step. In case of disagreement between the two analysts, the articles were evaluated by a third author (NG).

### 2.5. Step 4: Data Classification

Studies deemed relevant were organized into a data extraction table found in Table 2. The registered information included: title of the article; author and year of publication; type of the study; aim of the study; type of the instrument of mHealth; perioperative reference period; main features/use of the application; orthopedic field of reference; health professional involved; and benefits in the perioperative pathway. In the case of missing information, the label “not available” was reported within the data extraction table.

### 2.6. Step 5: Comparison, Summary and Presentation of the Results

Lastly, the classification of the data allowed the elaboration of a report of the evidence that emerged from our scoping review. The data elaborated in the model were then analyzed on the basis of the research question, using a qualitative analysis of the content.

## 3. Results

**Figure 1:**
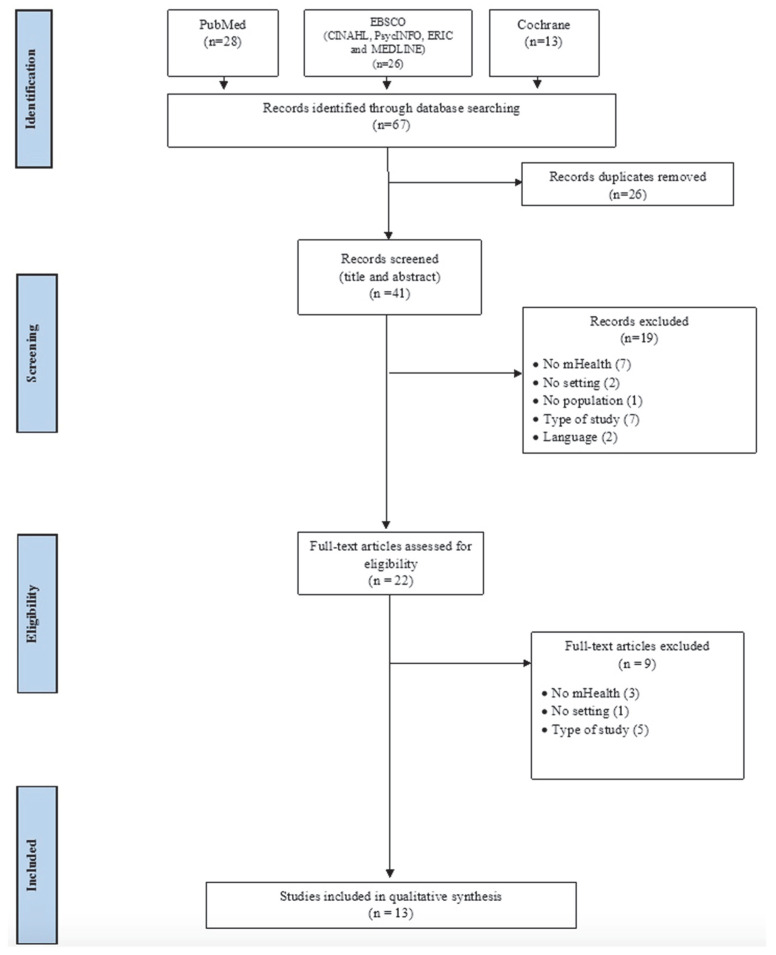
Flow diagram of the search and selection process, based on PRISMA flowchart.

A total of 67 articles were retrieved, 26 duplicates were removed, and of the remaining 41 studies, an additional 19 articles were excluded from the title and abstract analysis. In the eligibility phase, 22 articles were analyzed, of which 9 were excluded and 13 were found to be the final relevant studies (see Figure 1). The included studies were all in English.

One study is a non-randomized quasi-experimental design [24], three are cross sectional studies [20,25,30], five are observational studies [21,22,28,29,31], one is a pilot study [27], and three are RCT studies [19,23,26].

**Figure 2:**
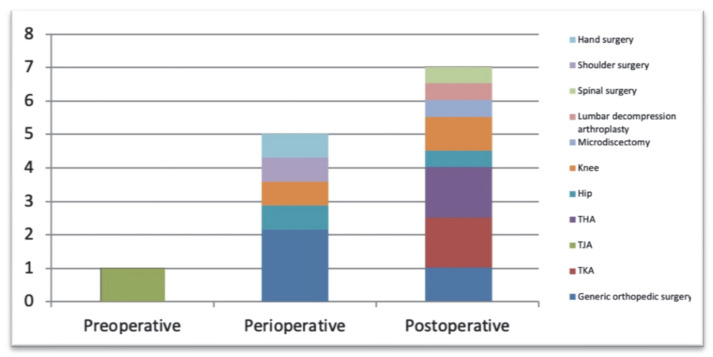
Main types of surgical interventions in relation to the operative period.

Most of the identified studies focus on the postoperative and perioperative management phase of surgical care, referring especially to major surgery. Figure 2 relates the operative period (pre, intra, and post) to the type of surgical procedures.

The results obtained were divided into two main categories of interest as follows:

### 3.1. Applications Oriented to Patient Management in the Perioperative Pathway

Most studies address the various aspects of patient management in the perioperative pathway, with greater emphasis on the postoperative period of orthopedic patient management.

Several studies, among those deemed relevant, have investigated how mHealth systems can be useful in controlling postoperative pain [19,20,21].

The study by Anthony and colleagues [19] shows that, in a total of 76 patients enrolled (38 in the intervention group and 38 in the control group), the use of mHealth systems for remote monitoring led to a decrease in opioid use. In fact, in the intervention group there was a 36.5% decrease in the use of opioid tablets. Moreover, subjects in the intervention group reported a lower postoperative pain intensity score (mean 45.9, SD 7.2) than subjects in the control group, (mean 49.7, SD 8.8; *p* = 0.04) [19].

The study by Premkumar and colleagues [20] investigates, again in relation to opioid use, how the use of an instant messaging platform improves communication between patient and surgeon about postoperative pain management. This platform helps capture daily information about opioid use in the post-surgery setting. Another study [21] evaluates how the use of cognitive-behavioral therapy, through an instant messaging platform, can ensure a better pain management in the postoperative period. Cognitive-behavioral therapy (CBT) is a common psychological intervention helping patients cope with chronic pain. In fact, it is reported that patients in the intervention group used less than the opioid prescribed daily (20%, IQR:10–27%) also with respect to controls (50%, IQR:4–68%).

Further related to the postoperative period, the study [22] evaluated how the use of mHealth systems is useful in controlling and reducing anxiety in the postoperative period. Specifically, diaries were used to record recurring themes over time, allowing the development of a library of messages to address postoperative anxiety. The pilot group consisted of 21 patients. The average rating of the application on a 1 to 5 scale with 5 being “very useful” was 4.57. Of the 12 patients available for postoperative interviews, 11 felt the content of the messages was relevant [22].

The study by Witting–Wells and colleagues [23] addresses a common issue for many patients in the post-surgery setting, therapeutic adherence. Specifically, an app was designed to remind patients to take anti-coagulant drugs. The study of van Dijk–Huisman and colleagues [24] focused on the physical recovery of patients undergoing orthopedic surgery, through the development of the Hospital Fit tool. Hospital Fit is designed for use in hospitalized patients and consists of a smartphone application connected to an accelerometer. The accelerometer algorithm was validated to differentiate lying and sitting from standing and walking in hospitalized patients. This application provides patients and involved professionals with feedback on the number of minutes spent standing and walking during the day [24].

The study by Pereira et al. [25] evaluated through the control of the knee rotation angle the postoperative follow-up. To do this, a knee goniometer based on a smartphone accelerometer was used. The study showed that smartphone goniometer was compatible with use in a clinical setting, provides relatively quick and easy measurements, and greater intra- and inter-observer reliability than the standard goniometer for single measurements.

Finally, the only study that focuses on the preoperative period was performed by Seward and colleagues [26], of which the focus of interest is weight reduction in patients undergoing total joint arthroplasty (TJA) major orthopedic surgery. To achieve the goal, study participants downloaded a smartphone application to follow a remote dietitian (RD) program. The dietitian contacted intervention participants weekly or biweekly via video calls and text messages for up to 3 months.

### 3.2. Applications Used by Healthcare Professionals

Among the different studies considered relevant, whose use is of sole relevance to health professionals, several studies focus on mHealth systems that allow, by sending of messages, a better communication between the different components of the team [27,28,29].

The study by Ebuluk and colleagues [27] explores how allowing different cases (totaling 283) to be discussed among different surgeons improves knowledge and communication. To do this, the mHealth pMD program was designed to provide secure messaging between healthcare teams, allowing colleagues to send and receive text, photo, and video attachments, which are all encrypted, keeping patient information secure. The group consisted of two private practice surgeons and three academic surgeons. Data were collected from 283 cases discussed during the study period. The mean number of reviewers who commented on a case was 2.4, with at least one response in 97% of cases. In 33% of the cases, the peers confirmed the initial treatment plan, and in 67% of the cases, an alternative treatment plan was recommended and executed. The case distribution was 94 primary and 189 revision procedures, including 173 hips, 103 knees, three ankles, two shoulders, and two pelvises [27]. Similar studies were performed by Khanna and colleagues [28] and Darawualla and colleagues [29]. The study by Khanna and colleagues [28] uses the WhatsApp^®^ platform to exchange information throughout the perioperative period between different members of the healthcare team and thus improve communication. The study by Darawualla and colleagues [29], on the other hand, uses the MyDoc application where a variety of functions are integrated, including a patient diary, virtual teleconsultations through a live videoconferencing system accessible from anywhere with an Internet or Wi-Fi connection, and a secure communication application.

The study of Macedo et al. [30], instead, focuses on the sharing of radiographic images to decide together with the team the surgical course of the subjects involved. The developed application, “*OrtopeX*” allows for the measurement of radiographic angles helping orthopedists in the therapeutic process. According to the analysis of perceived usefulness, 90% of residents responded positively to the questions, whereas among orthopedics the percentage was 75%, denoting a statistically significant difference (*p* = 0.002).

Finally, the study by Tulipan and colleagues [31] evaluates an intervention simulation application and how its use may affect the actual intervention. The application guides the user through the operations in a sequential manner. The user can practice an operation by taking a “tutorial” module and then take a “test” module to assess their understanding of the steps and techniques involved. The application does not teach the actual technical surgical skill needed to manage it, but rather aims to familiarize and prepare the user of a particular surgical procedure, the related medications and instruments, and the necessary surgical steps that the surgeon must perform. All cohorts, on average, improved their performance with each subsequent simulation attempt. For all attempts, the experts outperformed the novice and intermediate participants, while the intermediate cohort outperformed the novice cohort. Novice users consistently gave the app better scores for usefulness as a training tool, and demonstrated more willingness to use the product.

## 4. Discussion

The use of mHealth in orthopedic surgery has been shown to be very important in increasing patient involvement in the therapeutic process through better control and management of postoperative pain. Several studies have highlighted the importance of using SMS survey systems to collect patient-reported pain levels, opioid consumption, and adverse effects in the acute postoperative period after total knee arthroplasty (TKA), total hip arthroplasty (THA) and lumbar spine surgery [19,20,21]. The use of these systems showed high response rates and the possibility to capture granular data not seen with traditional phone, email, or mail surveys. Such information can be critical to improve patient counseling, deepen understanding of postoperative opioid use, and prompt new research questions as previously unknown trends are revealed [20].

Acute and chronic pain is influenced by psychological factors that ultimately result in interference with patients’ activities of daily living and decreased quality of life. Communication via mobile apps and text messaging with patients using the principles of cognitive behavioral theory has been shown to be useful and effective in decreasing opioid use and treating various psychological conditions and cognitive problems, including pain catastrophizing [21,22]. Patients view surgical outcomes differently than their physicians. In this context, using strategies to implement patient engagement and collect their perspectives and thoughts in detail are critical to help surgeons predict and improve the surgical outcomes of primary importance for patients, first of all pain control [19,20,21,22].

mHealth in orthopedic surgery in addition to post-operative pain management has also been found to be of great importance as a reminder for all those patients who have been prescribed anticoagulant therapy (ASA) for venous thrombosis prophylaxis using a real alarm on the phone, improving therapeutic adherence [23]. Early mobilization is also critical for good postoperative recovery in orthopedic surgery. In this regard, Hospital Fit has proven to be a good application for remote management of early mobilization. This application has an accelerometer attached to the upper leg, and the algorithm is able to differentiate between lying and sitting and standing and walking positions in patients using walking aids, or with slow or impaired gait [24].

The only study in this scoping review that focused on the preoperative phase was performed by Seward et al. [26], which reports the importance of adequate body weight to have a good postoperative outcome. Using an RD intervention and a mobile app aimed at helping patients become eligible for TJA. This study emphasizes the importance of frequent and verified weight measurements, which can be facilitated by the use of mHealth, implementing preoperative education and management for better postoperative outcomes.

Some relevant studies in this scoping review focus on the exclusive use of mHealth among healthcare professionals. In fact, one of the used tools is peer to peer mentorship, promoting active peer learning and providing countless opportunities to learn and collaborate with each other by asking advice from more experienced colleagues on decision making. Thus, having a positive impact on patient care has a very positive impact on reducing healthcare worker stress [27,32].

One of the main limitations of this scoping review is the reduced availability of studies on the use of mHealth systems in orthopedic surgery. On the other hand, the choice to include only studies published in English or Italian as specified in the methods section did not imply limitations in the availability of studies.

## 5. Conclusions

The mHealth allows us to personalize patient care, improve communication between professionals, and provide a technological advancement that facilitates remote care while reducing its costs [32,33]. In fact, the European Commission estimated already in 2017 that the use of these technologies would save about 99 billion euros, recommending its development [1].

As can be deduced from this scoping review, mHealth can have a major impact on the perioperative process inherent in orthopedic surgery. The use of messaging systems [19,20,21,22,29] through various software applicable to both Android and Apple systems can have a positive impact on patient participation in the therapeutic process, proper therapeutic adherence to opioid medications, reduction of anxiety, but above all facilitates communication between health professionals, increasing the quality of care. Applications [24,31] and alarms for cell phones [23] assume a fundamental role in mHealth applied to orthopedic surgery because they allow the user to be followed throughout the process, not only for surgical purposes but also for psychophysical recovery, the main feature that allows the applicability of these apps or alarms is the ease of use.

Finally, it is important to point out that, as shown by Wilkowska and Ziefle [34], data security and privacy are two important aspects for the successful use of mHealth systems by both patients and professionals. Special attention must be paid to the sharing of sensitive patient data, which must always be done in compliance with European regulations [35] or current privacy regulations.

## Figures and Tables

**Table 1:** Search strategy on PubMed.

Query in Pubmed
#11,“ ((((((“mobile health” [Title/Abstract]) OR (“mobile application *” [Title/Abstract])) OR (“mobile health application *” [Title/Abstract])) OR (“mHealth” [Title/Abstract])) OR (“mobile phone” [Title/Abstract])) OR (“smartphone” [Title/Abstract])) AND ((“orthopedic surgery” [Title/Abstract]) OR (“orthopedic care” [Title/Abstract]))”
#10,“orthopedic care [Title/Abstract]”
#9,“orthopedic surgery [Title/Abstract]”
#8,“mobile health” [Title/Abstract] OR “mobile application *” [Title/Abstract] OR “mobile health application *” [Title/Abstract] OR “mHealth” [Title/Abstract] OR “mobile phone” [Title/Abstract] OR “smartphone” [Title/Abstract]
#7, “smartphone [Title/Abstract]”
#6,“mobile phone [Title/Abstract]”
#5,“mHealth [Title/Abstract]”
#4,“medication management [Title/Abstract]”
#3,“mobile health application * [Title/Abstract]”
#2,“mobile application * [Title/Abstract]”
#1, “mobile health [Title/Abstract]”
The asterisk (*) is used to indicate the singular and plural of each word.

**Table 2:** Summary of findings.

N.	Author, Year	Type of the Study	Aim of the Study	Type of the Instrument of mHealth	Perioperative Reference Period	Orthopedic Field of Reference	Health Professional Involved	Benefits in the Perioperative Pathway
[19]	Anthony et al., 2020	Randomized controlled trial (RCT)	This study aims to evaluate the effects of ACT delivered via an automated mobile messaging robot on postoperative opioid use and patient-reported outcomes (PROs)	Acceptance and Commitment Therapy (ACT)	Post-operative	Orthopedic surgery	Orthopaedic surgeons	Reduction of post-operative pain
[20]	Premkumar et al., 2019	Cross sectional study (survey)	Improve the collection of information on patient-reported opioid use and evaluate use to treat postoperative pain	SMS text messaging platform	Post-operative	Total knee arthoplasty, total hip arthoplasty Hip, knee, microdiscectomy, and lumbar decompression arthroplasty	Orthopaedic surgeons	Proper management of opioid intake
[21]	Rojas et al., 2019	Case control study	Through psychological intervention improve postoperative pain	Cognitive behavioral therapy (CBT) through mobile messaging	Post-operative	Musculoskeletal tumors undergoing outpatient orthopedic surgery	Orthopaedic surgeons	Postoperative pain control and proper management of opioid and analgesic medications
[22]	Goz et al., 2019	Observational study	Reduce perioperative anxiety	Messaging application	Post-operative	Spinal surgery	Orthopaedic surgeons	Reduce anxiety
[23]	Wittig–Wells et al., 2019	Randomized controlled trial (RCT)	To evaluate the preliminary impact of a preset telephone alert on medication adherence in adults prescribed ASA for 35 days after knee or hip arthroplasty	Telephone alarm	Post-operative	Total knee arthoplasty, total hip arthoplasty	Orthopaedic surgeons	Improvement of therapeutic adherence in the follow-up of orthopedic patients
[24]	van Dijk–Huisman et al., 2020	Non-randomized quasi-experimental design	Use of mHealth tools to facilitate improved functional recovery, reduced length of stay, reduced pain, and low mortality rates in a controlled fast-track programa(TKA, THA)	Hospital Fit	Post-operative	Total knee arthoplasty, Total hip arthoplasty	Physiotherapists	Increased postoperative recovery and continuous monitoring of P.V. linked to mobilisation
[25]	Pereira et al., 2017	Cross-sectional reliability trial	To compare the reliability of a smartphone accelerometer-based knee goniometer versus a standard knee goniometer for active and passive knee ROM assessment	Smartphone accelerometer-based knee goniometer application	Post-operative	Knee surgery	Orthopaedic	Improved postoperative evaluation of patients
[26]	Seward et al., 2020	Randomized controlled trial (RCT)	The primary objective is to evaluate the feasibility and effectiveness of a 12-week weight loss intervention with diet and physical activity supervised by RD and a mobile app for patients with severe obesity prior to undergoing TJA	An online/smartphone telemedicine application(Nutrimedy, Brookline, MA, USA)	Pre-operarive	Total joint arthoplasty	orthopedic surgeons and dietitians	BMI reduction to perform the surgery
[27]	Elbuluk et al., 2018	Pilot study	Improving orthopedic surgery through peer-to-peer communication	Messaging systems	Perioperative	Hip, knee, pelvis, and shoulder	Orthopaedic surgeons	Sharing of information and consequently improvement of orthopedic surgery
[28]	Khanna et al., 2015	Observational study	Report the impact of the introduction of a smartphone application ‘‘WhatsApp’’ as an intradepartmental communication tool Communication tool on: awareness of patient information, efficiency of the handover process among orthopedic residents in a 300-bed tertiary teaching center and the duration of the traditional morning handover	WhatsApp	Perioperative	Orthopedic surgery	Orthopedists and residents	Improved communication among team members with improved perioperative management of patients
[29]	Daruwalla et al., 2014	Prospective study	Determine staff reaction to MyDoc and its secure mobile telemedicine application and alternative messaging platform in an orthopedic clinical setting in Singapore	MyDoc	Perioperative	Orthopedic surgery	Orthopaedic surgeons, orthopaedic assistants and residents	Provide a secure way through which patients can communicate with their key teams at a time and in a way that is convenient for both parties
[30]	Macedo et al., 2021	Cross sectional study (survey)	Evaluate a radiographic image analysis application for orthopedic physicians and orthopedic residents	OrtopeX application for radiographs and angle measurements comprise an essential mechanism in the diagnosis, treatment, planning, and evaluation of orthopedic surgery outcomes	Perioperative	Imaging radiographic	Orthopaedic surgeons	Rapid diagnosis
[31]	Tulipan et al., 2019	Cohort study	The primary purpose of this study was to evaluate the validity of the app and correlations between app performance and surgical skill level, as well as to determine whether practice with the simulator results in improved performance for participants	Touch Surgery	Perioperative	Not available	Orthopaedic surgeons, orthopaedic assistants and residents	This study provided direct evidence in the orthopedic literature that simulator training is directly transferable to operating room efficiency and effectiveness

## Data Availability

All data are available upon request.

## Supplementary Materials

The following are available online at https://www.mdpi.com/article/10.3390/ijerph182312549/s1, File 1.
